# Inanimate 3D printed model for thoracoscopic repair of esophageal atresia with tracheoesophageal fistula

**DOI:** 10.3389/fped.2023.1286946

**Published:** 2023-11-14

**Authors:** Petra Zahradniková, Jozef Babala, Rebeka Pechanová, Martin Smrek, Pavol Vitovič, Miroslava Laurovičová, Tomáš Bernát, Barbora Nedomová

**Affiliations:** ^1^ Department of Pediatric Surgery, Faculty of Medicine, Comenius University and National Institute of Children's Diseases, Bratislava, Slovakia; ^2^ Faculty of Medicine, Institute of Medical Education and Simulations, Comenius University, Bratislava, Slovakia; ^3^ Faculty of Medicine, Comenius University, Bratislava, Slovakia; ^4^ Department of Paediatric Anaesthesiology and Intensive Medicine, Faculty of Medicine, Comenius University and National Institute of Children's Diseases, Bratislava, Slovakia

**Keywords:** oesophagus, minimally invasive surgery (MIS), thoracoscopic surgery, oesophageal atresia (EA), laparoscopic training, synthetic tissue, models, simulation

## Abstract

**Background:**

Thoracoscopic repair of esophageal atresia (EA) and tracheoesophageal fistula (TEF) poses significant technical challenges. This study aimed to develop an inexpensive, reusable, high-fidelity synthetic tissue model for simulating EA/TEF repairs and to assess the validity of the simulator.

**Methods:**

By using 3D printing and silicone casting, we designed an inexpensive and reusable inanimate model for training in thoracoscopic EA/TEF repair. The objective was to validate the model using a 5-point Likert scale and the Objective Structured Assessment of Technical Skills (OSATS) to evaluate participants' surgical proficiency.

**Results:**

A total of 18 participants (7 medical students, 4 pediatric surgery trainees, and 7 experienced surgeons), after being instructed and trained, were asked to perform TEF ligation, dissection, as well as esophageal anastomosis using six sliding knots on the EA/TEF simulator. All participants in the expert group completed the task within the 120-minute time limit, however only 4 (57%) participants from the novice/intermediate completed the task within the time limit. There was a statistically significant difference in OSATS scores for the “flow of task” (*p *= 0.018) and scores for the “overall MIS skills” (*p* = 0.010) task distinguishing between novice and intermediates and experts. The simulator demonstrated strong suitability as a training tool, indicated by a mean score of 4.66. The mean scores for the model's realism and the working environment were 4.25 and 4.5, respectively. Overall, the face validity was scored significantly lower in the expert group compared to the novice/intermediate groups (*p* = 0.0002).

**Conclusions:**

Our study established good face and content validity of the simulator. Due to its reusability, and suitability for individual participants, our model holds promise as a training tool for thoracoscopic procedures among surgeons. However, novices and trainees struggled with advanced minimally invasive surgical procedures. Therefore, a structured and focused training curriculum in pediatric MIS is needed for optimal utilization of the available training hours.

## Introduction

Pediatric surgeons have shown growing interest in minimally invasive surgery (MIS) for esophageal atresia with distal tracheoesophageal fistula (EA/TEF) since the initial successful thoracoscopic repair performed by Rothenberg in an infant with EA/TEF (1). Performing thoracoscopic repair for EA/TEF poses technical challenges and remains an unfamiliar procedure for numerous pediatric surgeons. Furthermore, the majority of infants undergoing EA/TEF repair are exceptionally small at the time of surgery, resulting in a confined and narrow surgical field that presents considerable technical difficulties for the surgeons. MIS in infants involves a challenging learning curve when it comes to suturing techniques and tissue manipulation. This learning curve often translates to extended operative times until surgeons attain full expertise in these skills (2). Specifically, suturing techniques pose significant challenges, especially in pediatric patients, because of the smaller tissue size and the restricted abdominal space for maneuvering instruments (3). On the other hand, pediatric surgeons often spend a significant portion of their careers waiting to perform certain procedures. The stakes with pediatric patients are so high that usually only the most experienced specialists are allowed to perform these procedures, creating a gap. Thus, a gap is created when experienced surgeons in the middle of their careers become frustrated while realizing they have so much further left to go. They realize they are expected to sit and wait until it is finally their turn.

Simulation has proven to be a fundamental tool in surgical education (4). In recent years, shortening of the learning curve has been demonstrated by implementing of wide range of surgical models (5–7). Due to the possibility of repeating the simulation, both the surgeons and residents have the great opportunity to learn surgical techniques, or particular steps, with the possibility of making mistakes, practice abilities and procedures in standardized and supervised situations (8). Ideally, simulation should enable the deliberate, repetitive, and participatory practice of neonatal MIS operative steps. This practice should utilize a validated model capable of identifying and correcting performance errors (9–11). Similarly, it should determine when the required skills have been acquired with a reliable degree of accuracy (12). The integration of three-dimensional (3D) printing technology and imaging data from CT and MRI scans has unveiled new possibilities in crafting high-fidelity laparoscopic simulators. These simulators accurately replicate, to scale, the environment encountered in neonatal surgery (10). This technology is potentially more cost-effective and avoids the ethical issues associated with using cadaveric and animal tissues. It is believed that due to current technological advances the future models will likely be both of high fidelity and low cost (12). Conversely, inanimate simulation models, especially those considered low-fidelity, are thought to lack the realism found in more sophisticated counterparts. However, research suggests that increased realism in a simulator doesn't necessarily correlate with improved learning outcomes. This prompts questions about the justification of the added costs associated with high-fidelity simulators, especially when comparable knowledge and skill outcomes can be attained with more budget-friendly alternatives (13, 14). The objective of this study was to introduce and validate an inexpensive, reusable inanimate model designed for training in the thoracoscopic repair of EA/TEF.

## Methods

### Simulator development

**Figure 1:**
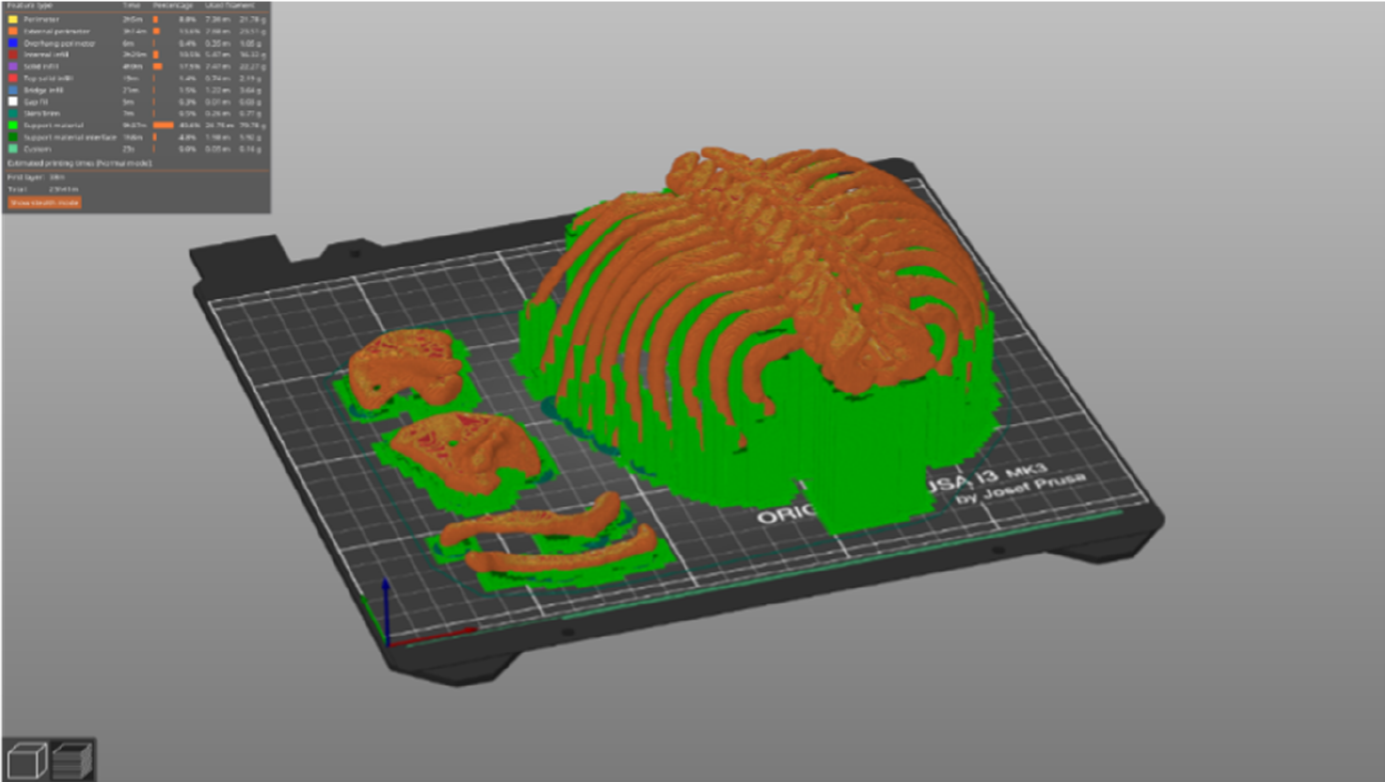
Process of segmentation: CT scan was utilized to segment a child's spinal column along with the ribcage, scapula, and clavicles using 3D slicer.

**Figure 2:**
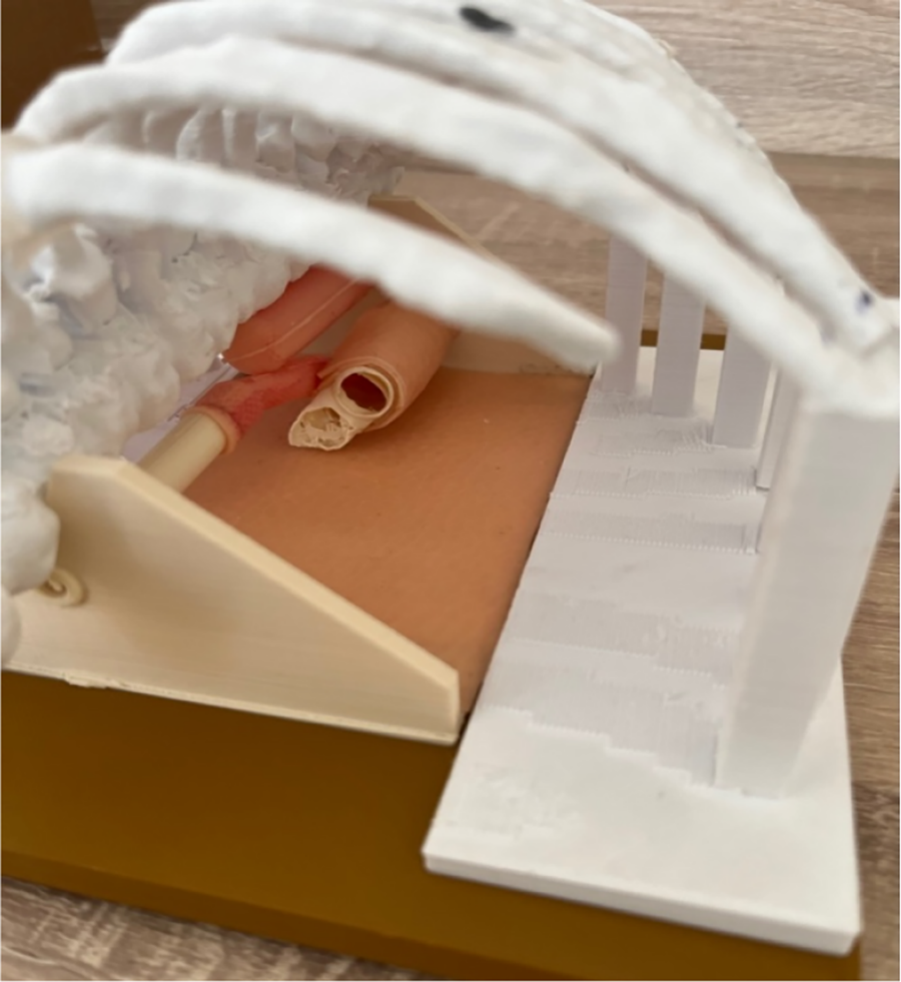
3D-printed components consist of three models: a baseplate, to which the other two parts securely attach to prevent any unwanted movement, a ribcage model, and an internal fixator model.

**Figure 3:**
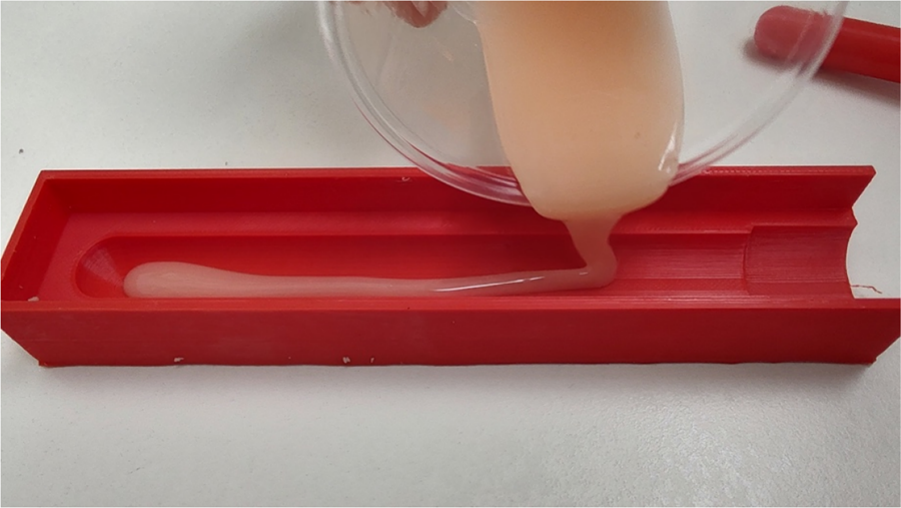
For crafting the silicone esophagus, an FDM printer was also employed to produce two molds made of PLA plastic—one for the distal pouch and another for the proximal one. The diameter of the proximal esophagus was 10 mm, while the distal esophageal pouch measured 7 mm in diameter.

**Figure 4:**
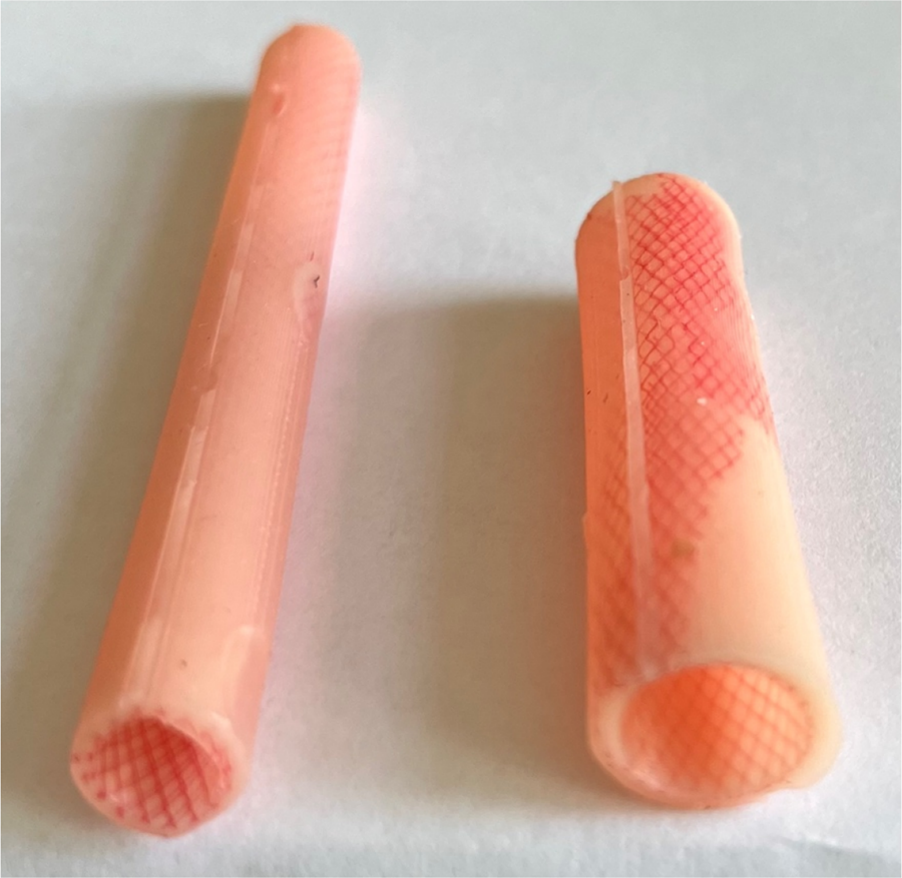
Esophageal stumps are created using silicone, which is poured into a mold generated by a 3D printer. We determined the oesophagus's diameter by measuring the actual width from the initial x-ray image, with a nasogastric tube inserted.

**Figure 5:**
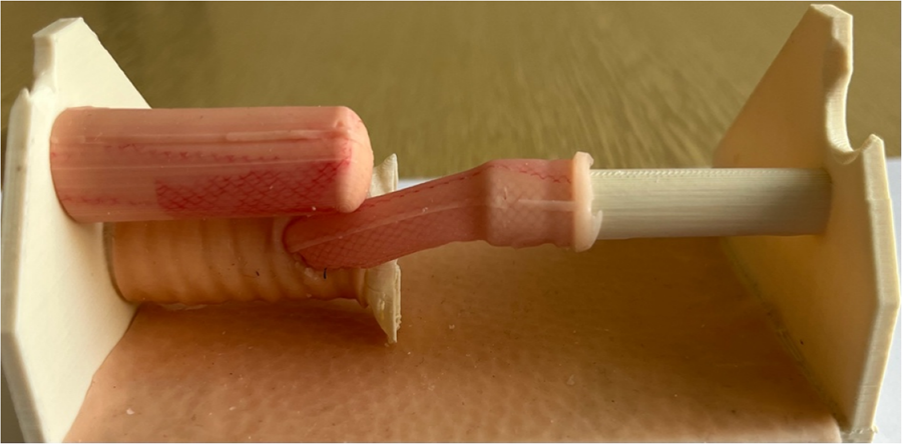
Internal part EA/TEF model ready for training. Silicone esophagus can be changed by one person in 5 min.

**Figure 6:**
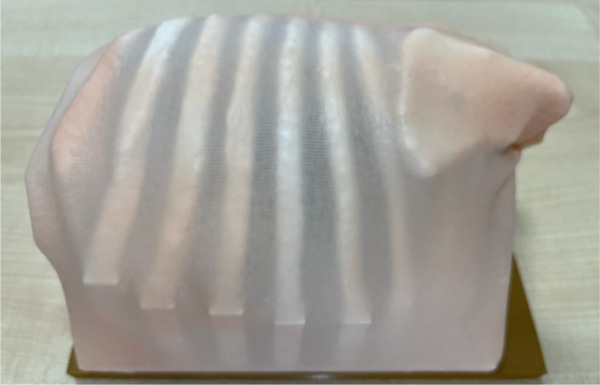
The surface of the chest model covered with silicone skin.

The esophageal atresia model was developed using a combination of FDM (fused deposition modeling) 3D printing and casting of platinum-cured silicone. Initially, a CT scan was utilized to segment a child's spinal column along with the ribcage, scapula, and clavicles using 3DSlicer. The resulting STL file (a format for saving 3D models) was then modified in Blender3D to incorporate internal fixtures for the esophageal pouches and trachea (Figure 1). In total, the 3D-printed components consist of three models: a baseplate onto which the other two parts securely attach to prevent any unwanted movement, a ribcage model, and an internal fixator model (Figure 2). The printing process took slightly less than 24 h to complete using a Prusa i3 MK3S printer set to a layer height of 200 micrometers, with the spinal column positioned facing upwards. A total of 178 g of Prusament PLA (priced at 30 Euro per kilogram) was used, resulting in a 3D filament cost of 5.00 Euro. For crafting the silicone esophagus, an FDM printer was also employed to produce two molds made of PLA plastic—one for the distal pouch and another for the proximal one. These molds, when filled with silicone, enabled the creation of a hollow tube with a wall thickness of under 2mm. The silicone was prepared by blending two components (A and B) along with a designated silicone dye (Figure 3). Power-mesh was integrated into the silicone mixture to prevent tearing during suturing. The curing process of this silicone takes up to four hours, which meant that several days were required to complete the task and produce an adequate number of esophagus models and pouches (Figures 4, 5). To simulate human skin, the same silicone and dye were used, poured loosely into a rectangular mold (25 × 25 cm) with a height of 1 mm and lined with power-mesh. The bottom surface of the mold was composed of synthetic leather, which imprinted a skin-like texture onto the skin imitation (Figure 6).

### Participants

The current study was conducted between October 2022 and August 2023 at the National Institute of Children's Diseases in Bratislava, Slovakia. All participants were anonymized and categorized as novice, intermediate, and expert based on their previous training and experience levels. Novice participants were defined as medical students in their fifth year of medical study. Intermediate participants were identified as pediatric surgical trainees in the 4th, 5th and 6th years of training. Expert participants were characterized by having a minimum of 15 years of experience in pediatric surgery. Prior to attempting the trial, every participant viewed brief instructional videos and accompanying written guidelines for each task. During each trial, participants were also given straightforward instructional cues. Furthermore, simple instructional cues were reiterated throughout each trial. The tasks included:

a) Closure of TEF using a single sliding knot suture
b) Division of TEF
c) Opening of the upper esophageal pouch
d) Esophageal anastomosis involving three sliding knots on the back side and three on the front side. The anastomosis was performed intracorporeally in simulator All participants' attempts at these tasks were recorded and saved for subsequent analysis.

Throughout the procedures, SZABO-BERCI-SACKIER Laparoscopic Trainer was used. This laparoscopic trainer contains diaphragms at the typical puncture sites and a flexible endoscope holder that provides the surgeon with the ability to manipulate instruments with both hands. This trainer was borrowed for the purpose of this study from the RADIX MEDICAL Ltd. team (exclusive distributor of KARL STORZ in Slovak republic).

### Assessments and parameters examined

After each procedure, four parameters were assessed:

• **Surgical time**: This metric measured the time taken from the entry of instruments into the simulator until the completion of the last sliding knot.
• **Objective structured assessment of technical skills (OSATS):** Performance during the simulated tasks was evaluated using the OSATS methodology. Each individual trial was recorded and later reviewed by three independent expert pediatric surgical consultants who were blinded to the identity and skill level of each participant. A modified OSATS scoring system was utilized to grade each trial on various aspects, including dexterity, flow of task, spatial orientation, needle holding, instrument movement, sliding knots performing, MIS skills, and overall performance. Scores ranged from 1 (inexperienced) to 5 (expert) for each aspect, resulting in a total score range of 8–40. The examiners maintained their blindness to participant identity and skill level throughout the assessment process.
• **Participants' feedback:** A Likert scale questionnaire was employed to gather participant feedback, with responses ranging from 1 to 5, addressing the following parameters:
  • Face validity
    a) Realism of the model
    b) Haptic feedback on materials
  • Content validity: Assessing the value and suitability of the model as a training tool.
  • Overall impression
    a) Utility as a simulator
    b) Utility for training in thoracoscopic EA/TEF repair
    c) Utility as training and educational tools
• **Quality of the anastomosis:** This aspect involved a blinded analysis in each model, evaluating the number of sufficient knots, luminal passage, and anastomosis strength under tension.

**Figure 7:**
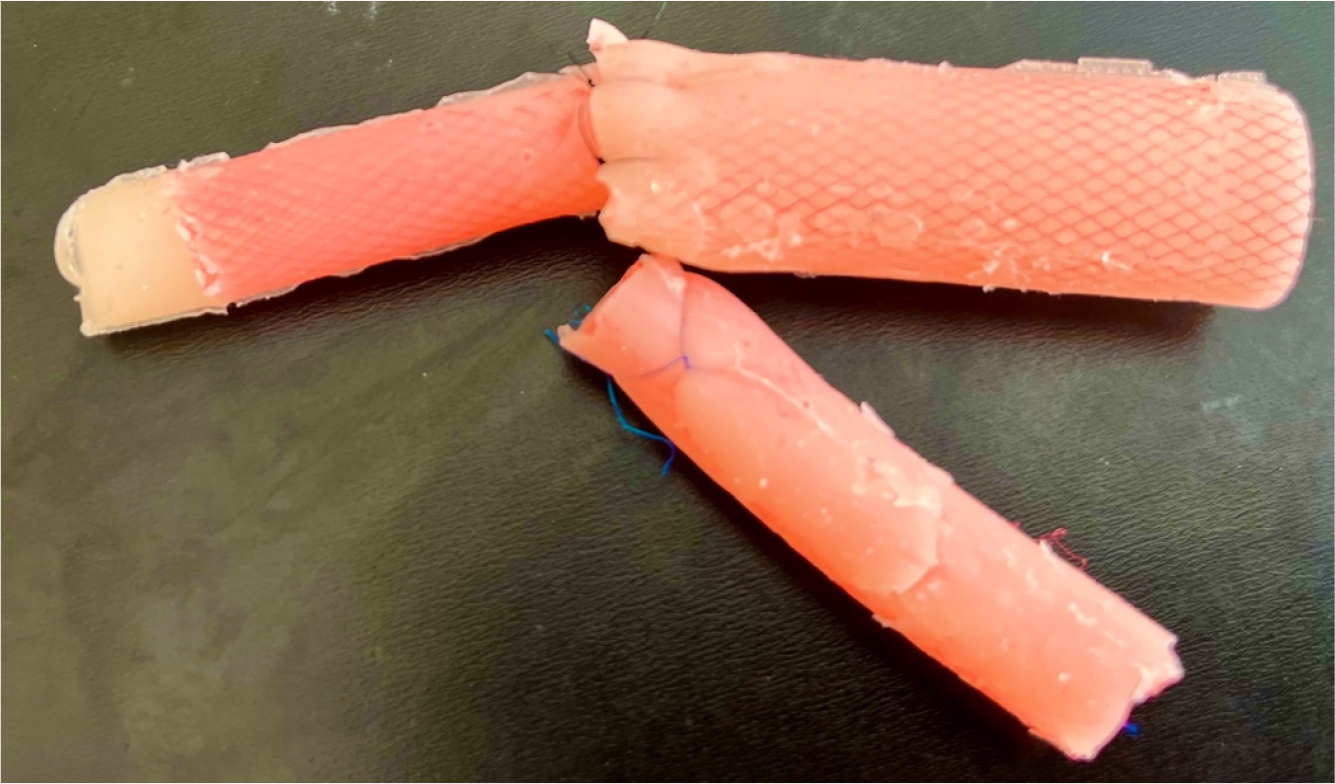
Model prepared for analysis.

**Figure 8:**
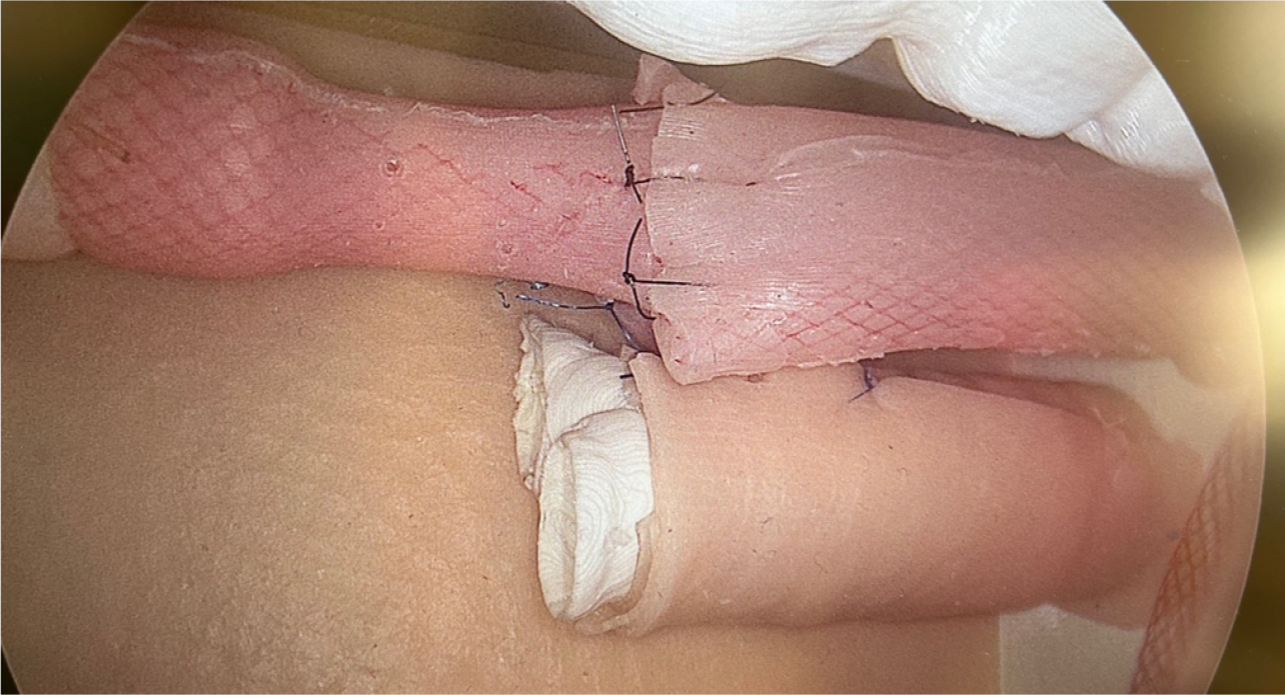
EA/TEF model in simulator (after proximal and distal pouches anastomosis).

Each model was reviewed by two independent expert pediatric surgical consultants who were blinded. The goal of this analysis was to assess the number of sufficient knots (the task involved creating 6 knots around EA). Luminal passage evaluation was performed using flexible endoscopy. To measure the strength of the anastomosis under tension, we employed a spring force meter, applying a tensile force of 250 g (equivalent to 2.5 N). We deemed the anastomosis successful if it could withstand a tensile force of 2.5 N (Figures 7, 8).

### Trial study

**Figure 9:**
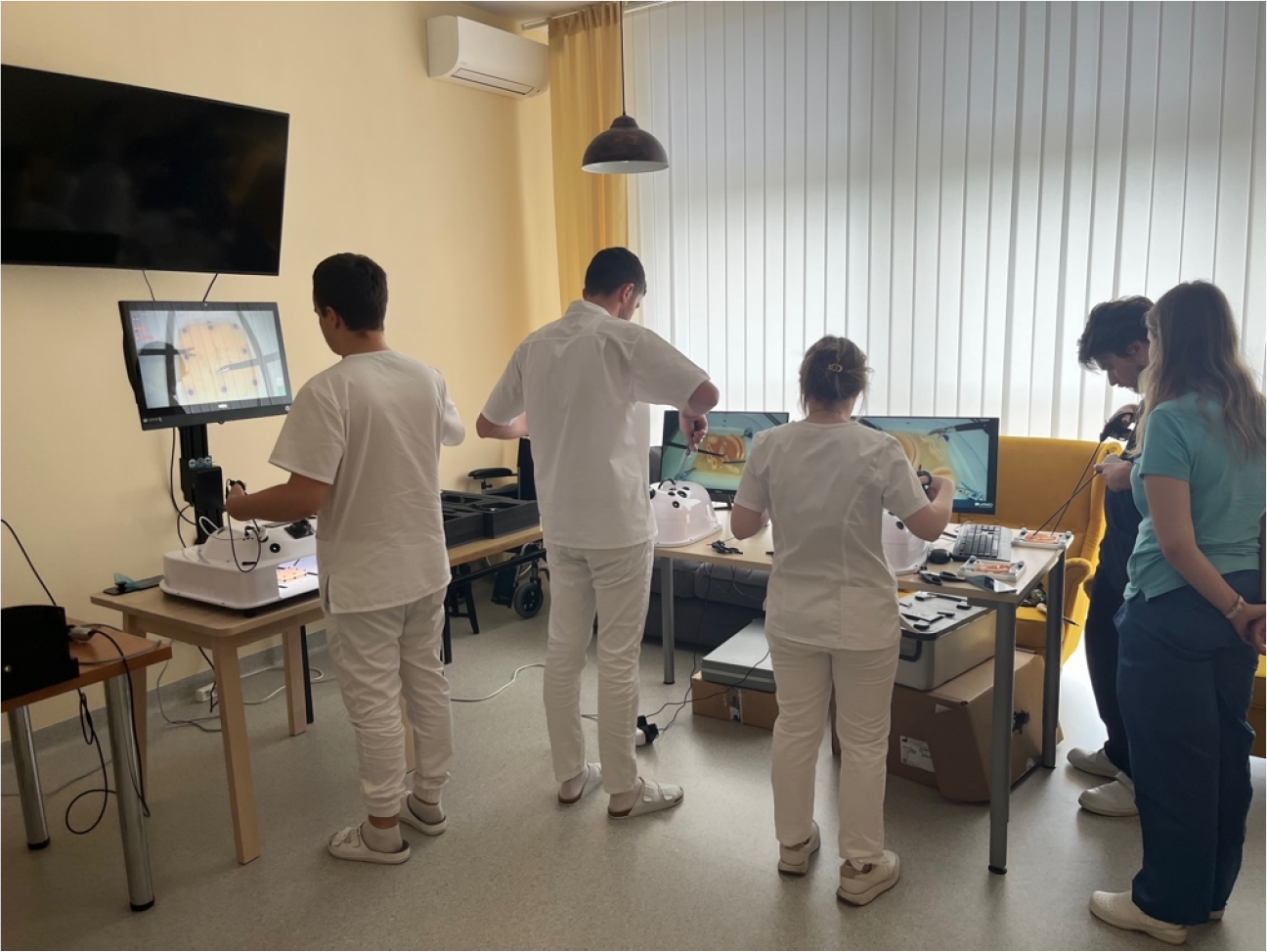
Basic/advanced laparoscopic training.

Initially, each participant was allowed to train basic and advanced laparoscopic skills (needle holding, sliding knot suturing) according individually experience. This training was tailored to individual experience levels. Laparo Adavance (LAPARO Sp. z o.o. Poland, European Union) and Laparo Analytic (LAPARO Sp. z o.o. Poland, European Union) simulation tool was used for training (Figure 9).

Throughout the procedures, Karl Storz laparoscopic equipment and recording devices were utilized. The surgical instrument setup included a 5-mm telescope at a 30-degree angle and 3-mm instruments (grasper, needle holder, and scissors). Surgipro 5/0 (polypropylene) thread was used for the continuous sliding knot sutures for the EA/TEF repair. Standard port placement procedures were followed. Each task commenced by initiating computer video recording and inserting a 30° 5 mm laparoscopic camera into the simulator. Subsequently, participants introduced their specific tools through standardized pre-made punctures in the silicone skin cover. The model was utilized to simulate the thoracoscopic EA/TEF repair. Only the internal silicone pouches required replacement after each use, incurring a cost of approximately 5 Euros per participant. Between participants, the model could be prepared for reuse in less than 10 min.

### Statistical analysis

Statistical analyses were performed with SPSSv25 software (IBM, NY, USA). All values were represented as mean with the standard deviation. The Mann–Whitney *U* test and one-way ANOVA were used to ascertain significant differences between groups. A nominal significance level of 0.05 was adopted.

## Results

The study received approval from the hospital ethics committee. Eighteen participants were enrolled in the study, with a median age of 33.5 years (interquartile range: 20–59). Among the participants, 7 (39%) were female, and 17 (94%) were right-hand dominant. The participant distribution included 7 (39%) medical students classified as novices, 4 (22%) intermediate (pediatric surgery trainees with fewer than 20 minimally invasive surgeries of experience), and 7 (39%) experienced individuals with over 15 years of expertise in pediatric surgery. In the expert group, all participants successfully completed the task within the allocated 120-minute timeframe. However, only one participant from the novice group and 2 (50%) form the intermediate group managed to complete the task within the time limit. Notably, the time taken to complete the attempt was longer in the intermediate and novice groups compared to the expert group (*p *= 0.006). Five (71%) medical students managed to finish the task involving TEF closure with a single sliding knot. However, they lacked the skill to perform end-to-end anastomosis using sliding knots. In the group of inexperienced surgeons, 2 (50%) were able to complete all three tasks (Table 1).

**Table 1:** Participant characteristics.

Level of training/expertise	Total number of participants	Mean age year (SD)	MIS procedures/year	Completed TEF ligation and division	Completed all 3 tasks within time limit (120 min)	Mean operating time (min)
Novice	7	22 (0.95)	0	5 (71%)	1 (14%)	142 (101–188)
Intermediate	4	30 (0.95)	<20	4 (100%)	2 (50%)	93 (70–109)
Expert	7	48.5 (6.16)	>20	7 (100%)	7 (100%)	73 (57–95)

All participants in the expert group managed to complete all three tasks, however, when examining the quality of the anastomosis, it was revealed that only 5 (71%) models in the expert group had a sufficient number of knots. The adequacy of anastomosis strength under tension was observed in 4 (57%) models, whereas one anastomosis was too tight for effective lumen passage.

### Quality of the anastomosis

A total of 10 models with esophageal anastomosis were evaluated. Among these models, there were 1 novice, 2 intermediate, and 7 experienced participants. Out of the 10 models assessed, only 5 demonstrated sufficient anastomosis with 6 stitches each, and all of these models were from the expert group. In the case of the other models, either the stitches were missing before the evaluation, or they had become loosened (untied) during the assessment. The adequacy of anastomosis strength under tension was observed in 4 models (using tensile force of 2.5 N), whereas one anastomosis was too tight for effective lumen passage (using flexible endoscope). Nine models could be evaluated by passage with a flexible endoscope (luminal passage was possible).

Experienced surgeons achieved a higher total OSATS score compared to the inexperienced group, consisting of novices and intermediates (18 (15–23) vs. 15.6 (7–22), *p* = 0.313); however, this difference did not reach statistical significance. Notably, there was a statistically significant discrepancy in OSATS scores for the 'flow of task' (*p* = 0.018) and 'MIS skills' (*p* = 0.010) tasks, effectively distinguishing between novices/intermediates and experts. While experts tended to score higher than novices and intermediates for each task, this trend did not achieve statistical significance, likely reflecting the study's statistical power (Table 2).

**Table 2:** OSATS scoring results.

		Novices (*n* = 7)	Intermediate (*n* = 4)	Expert (*n* = 7)	*p-*value
Dexterity	Median (range)	2 (1–3)	2.75 (2–3)	2.3 (2–3)	0.209
Flow of task	Median (range)	1.86 (1–3)	3.25 (3–4)	2.57 (2–4)	0.018
Spatial orientation	Median (range)	2.23 (1–4)	3 (2–4)	3.14 (2–4)	0.230
Needle holding	Median (range)	2.14 (1–3)	2.5 (1–4)	2.71 (2–4)	0.579
Instruments movement	Median (range)	1.85 (1–3)	2.75 (2–3)	2.71 (2–4)	0.100
Sliding knots	Median (range)	1.57 (1–3)	2 (1–3)	1.71 (1–3)	0.684
MIS skills	Median (range)	1.42 (1–3)	2.5 (2–3)	2.57 (2–3)	0.010
Overall score	Median (range)	13 (7–22)	18.75 (15–22)	18 (15–23)	0.074

### Content validity

Using a 5-point Likert scale, the majority of participants expressed the view that the 3D synthetic model of EA/TEF serves as a suitable training tool (mean: 4.68). Almost all participants believed there is a role for simulation-based training and educational training (mean: 4.62). When evaluating the content validity of TEF dissection/ligation and sliding knot anastomosis between esophageal pouches, the experienced group provided realistic mean scores of 4.12 and 4.14, respectively.

### Face validity

For assessing face validity, the experienced group responded to a series of questions aiming to rate the realism of different aspects of the simulator using a 5-point scale. The mean scores for the realism of the model and the working environment were 4.25 and 4.5, respectively. The haptics of the esophageal pouches received the lowest score (3.75). Interestingly, the experienced group scored significantly lower than the novice /trainee's group for 'TEF caliber' (3.57 vs. 4.22, *p* = 0.002) and 'proximal esophageal pouch caliber' (3.71 vs. 4.55, *p* = 0.003). Overall, the face validity was scored significantly lower in the experienced group compared to the inexperienced group (*p *= 0.0002). In terms of the overall impression, the highest ratings in the physical attribute's domain were for the overall impression and the usefulness of the tool as a simulator, with mean scores of 4.66 and 4.75, respectively. A comprehensive breakdown of responses from all participants can be found in Table 3.

**Table 3:** Model validation: results from the Likert Scale Survey (*n* = 17).

	Novices/intermediate (*n* = 11)	Expert (*n* = 7)	Overall (*n* = 18)	*p-*value
Face validity (mean score [SD])	4.44 (0.61)	3.8 (0.79)	4.16 (0.79)	0.0002
Realism of the model (mean score [SD])	4.33 (0.4)	4.14 (0.57)	4.25 (0.83)	0.916
3D neonatal thorax model (working environment) (mean score [SD])	4.66 (0.51)	4.28 (0.95)	4.5 (0.71)	0.07
Proximal esophageal pouch caliber (mean score [SD])	4.55 (0.69)	3.71 (0.75)	4.18 (0.78)	0.003
Distal esophageal pouch caliber (mean score [SD])	4.44 (0.70)	3.57 (0.53)	4.06 (0.78)	0.035
TEF caliber (mean score [SD])	4.22 (1.15)	3.28 (0.48)	3.81 (1.05)	0.002
Haptic feedback on materials (mean score [SD])	4.13 (1.03)	3.77 (0.75)	3.97 (0.93)	0.05
General appearance (mean score [SD])	4.33 (1.07)	4 (0.75)	4.17 (0.94)	0.015
3D neonatal thorax model (working environment) (mean score [SD])	4.33 (0.84)	4.14 (0.89)	4.25 (0.84)	0.285
Proximal esophageal pouch (mean score [SD])	3.88 (1.23)	3.57 (0.78)	3.75 (1.04)	0.035
Distal esophageal pouch caliber (mean score [SD])	3.88 (1.10)	3.57 (0.78)	3.75 (0.97)	0.003
TEF caliber (mean score [SD])	4.22 (0.82)	3.57 (0.53)	3.93 (0.79)	0.836
Content validity (mean score [SD])	4.52 (0.89)	4.28 (0.78)	4.41 (0.84)	0.003
Value and suitable as a training tool (mean score [SD])	4.77 (0.67)	4.57 (0.54)	4.68 (0.66)	0.264
TEF dissection and ligation (mean score [SD])	4.33 (0.96)	4.12 (0.89)	4.25 (0.91)	0.090
EA anastomosis (mean score [SD])	4.44 (1.05)	4.14 (0.89)	4.31 (0.97)	0.039
Overall impression (mean score [SD])	4.70 (0.31)	4.62 (0.53)	4.66 (0.43)	0.07
Useful as simulator (mean score [SD])	4.88 (0.31)	4.57 (0.54)	4.75 (0.44)	0.061
Useful as thoracoscopic EA with TEF training tool (mean score [SD])	4.66 (0.51)	4.57 (0.53)	4.63 (0.51)	1.000
Useful as training and educational tool (mean score [SD])	4.55 (0.51)	4.71 (0.48)	4.62 (0.49)	0.529

## Discussion

Across the globe, there is a growing adoption of MIS techniques in pediatric surgery, with these methods becoming the preferred standard for specific indications. As a result, there is an increasing demand for laparoscopic surgeons who are not only well-trained but also certified to address this expanding requirement (15, 16). MIS poses greater technical challenges compared to conventional open surgery, with a heightened risk of complications, particularly during the initial stages of experience (17). The demand for pediatric surgical simulators appears to be increasing as a consequence of the rarity of several complex neonatal conditions (e.g., EA), and the limited opportunities the trainees have to acquire the demanded technical skills on actual neonates. Simulation can be of low or high fidelity, according to how closely it resembles real life (10). The degree of fidelity should align with the specific task and the stage of training that the participant is in (11). The combination of three-dimensional (3D) printing technology and imaging data from CT and MRI scans has created new possibilities for developing high-fidelity laparoscopic simulators. These simulators can accurately replicate, to scale, the environment encountered in neonatal surgery (12). Nair et al. created and validated with using 3D modeling and printing high fidelity model with the exactly size and shape of neonatal chest. They demonstrated construct validity for two basic “foundation” tasks: ring transfer and needle pass. They concluded, that complex tasks such as intracorporeal suturing will be needed for training and assessing more experienced surgeons (18). Our study showcased that in more intricate tasks, such as intracorporeal suturing involving sliding knots, the capability to distinguish between medical students and trainees, as well as between trainees and experts, was particularly effective. It's plausible that surgeons in training already possess good spatial awareness and experience in minimally invasive surgeries; however, they may lack proficiency in intracorporeal suturing. Simulation offers the advantage of being conducted in a low-risk setting outside of the operating theater, eliminating any potential harm to patient safety. Participants can utilize validated models to practice specific tasks or the entire procedure in a deliberate and repetitive fashion within a secure and cost-effective learning environment (19). Assessing surgical skills in an objective manner presents challenges. Previous research has documented validated simulator metrics, encompassing factors such as time elapsed, the overall count of movements, and the cumulative path length. These metrics were subject to objective evaluation through specialized systems integrated within a simulator environment (20, 21). These metrics are valuable and can be readily evaluated through simulators. However, these assessments are cumulative in nature, aiding trainees in developing a tangible comprehension of their technical skills (22). Subjective validation methods involve gathering participant opinions, whereas objective measures are commonly employed in experimental studies. Subjective methods typically include face, content, referent, and expert validity, requiring participants to complete surveys about their experience with the model. On the other hand, objective approaches encompass construct, discriminative, concurrent, criterion, and predictive validity (23). It is widely acknowledged that simulators must undergo validation prior to their successful integration into educational initiatives. Wells et al. documented the development of a fully synthetic simulator for Thoracoscopic Esophageal Atresia/Tracheo-Esophageal Fistula. They noted that with each design iteration, the model's fidelity and validity improved. In their final version of the simulator, the recorded average scores were as follows: value as a training tool (4.8), relevance (4.6), physical attributes (4.5), realism of material (4.25), realism experience (4.17), and ability to perform tasks (3.77) (24). "We employed a commonly used 5-point Likert scale for the validation of our model. The calculated mean overall impression score was 4.6, indicating a generally positive perception across all participants. However, there was no statistically significant difference observed between the study groups concerning this overall impression score.

Conversely, we identified a significant disparity between the study groups in terms of face validity and content validity. This discrepancy underscores that the groups displayed contrasting views regarding the relevance and accuracy of the evaluation.

In summary, although no substantial divergence was noted between the study groups in relation to the mean overall impression score, we did observe significant variations concerning face validity and content validity. These findings provide valuable insights into how the study groups perceived the evaluation and its alignment with its intended purpose. The model received favorable assessments from a group of young and enthusiastic pediatric surgeons, as well as medical students with an interest in enhancing their minimally invasive surgical skills. High ratings were assigned to its usefulness as both a simulator and an educational tool for training purposes. The hands-on session was deemed very beneficial, with strong recommendations from the participants to other pediatric surgeons. The results from face and content validity assessments suggest that our 3D inanimate model of EA/TEF is likely to be well-received by both experts and trainees as an effective training tool. It is recommended that future studies delve into the model's training capabilities. Given its cost-effectiveness, reusability, and easy replacement for each participant, our model appears to hold significant potential as a thoracoscopic training device for surgeons. Simulation-based assessment is a crucial aspect of surgery, aiding in the acquisition of new skills and the achievement of improved surgical outcomes. The OSATS score facilitates a detailed analysis of surgical success (25, 26). The tool in question has achieved widespread usage and seeks to minimize the biases associated with subjective performance assessment. In this study, the tool was adapted to focus on specific aspects including dexterity, task flow, spatial orientation, needle handling, instrument movement, sliding knots performing, MIS skills and overall thoracoscopic skills. The results obtained from various participants exhibited similarities, but the most experienced surgeon, who had accumulated more hours of minimally invasive surgical training and conducted more monthly laparoscopic procedures, achieved the highest outcome.

“While a trend was observed for experts to achieve higher scores than novices and intermediates in each task, this difference did not attain statistical significance. This lack of significance is likely attributed to the restricted training on simulators within the experienced group.” There was a statistically significant difference in OSATS scores for the “flow of task” and scores for the “MIS skills” task distinguishing between novice and intermediates and experts. Nair et al. developed a fully synthetic 3D printed neonatal thoracoscopic simulator and aimed to establish its construct validity. By employing the OSATS score, they assessed three thoracoscopic tasks: ring transfer, needle pass, and incision of a blind upper esophageal pouch (EA cut). Within the group of 23 participants, these tasks were particularly effective in distinguishing between novices and experts, as well as novices and intermediates. However, the differentiation between intermediates and experts was not as pronounced. It's plausible that 'intermediate' surgeons already possess a strong spatial awareness and a substantial level of skill in needle manipulation. It could be reasonable to assume that more complex tasks, such as intracorporeal suturing, might be better suited for distinguishing between intermediate and expert surgeons (18). Several models for training in minimally invasive surgery (MIS) esophageal atresia (EA) repair have been documented in the literature (13). The majority of these models are inanimate, but there is an exception with the model described by Barsness et al., which involved an inanimate casing combined with fetal bovine tissue to replicate the organs involved in EA with tracheoesophageal fistula (TEF) repair (27, 28). Maricic et al. developed an inanimate and affordable model for training in minimally invasive surgery (MIS) for repairing esophageal atresia with tracheoesophageal fistula (EA/TEF). They found a correlation between the surgeon's prior experience and their performance in the model. This correlation was observed in terms of factors such as operating time, quality of anastomosis, and peripheral tissue damage (29). Barness et al. developed computer-aided drawings (CAD) to design a synthetic and appropriately sized model for esophageal atresia with tracheoesophageal fistula (EA/TEF). Their study concluded that this simulator can effectively train pediatric surgeons, particularly those in the early stages of their learning curve, in performing thoracoscopic EA/TEF repairs.

Nevertheless, our study does possess certain limitations. Primarily, the model was evaluated by a relatively small number of participants, a common concern observed in trials involving pediatric simulators. This issue is emphasized in the systematic review of simulation in pediatric surgery (30). Another limitation to acknowledge is that the study was conducted exclusively at a single center. Although this was sufficient to establish statistical significance between the groups, it should be noted that the experienced surgeon group was more accustomed to open EA/TEF procedures. the experienced surgeon group was more accustomed to open EA/TEF procedures. In the intermediate group, none of the participants had prior experience with thoracoscopic TEF repair in the past. In the expert group, 2 experts performed thoracoscopic TEF repair in the past, and all had experience with advanced minimally invasive surgery (MIS) in children and open EA/TEF procedure.

In conclusion, through a process of continuous improvement, the study successfully showcased the value of a high-fidelity simulation model for thoracoscopic esophageal atresia/tracheoesophageal fistula repair. Participants from various expertise levels, including medical students, trainees, and experts, found the simulator to be realistic and relevant for pediatric surgical training. The study also identified areas where the model could be enhanced, and these areas will be the focal points for future refinements of the synthetic EA/TEF repair simulator.

## Data Availability

The raw data supporting the conclusions of this article will be made available by the authors, without undue reservation.
